# Supplementary material for: MSBOTS: a multiple small biological organism tracking system robust against non-ideal detection and segmentation conditions
Source: PeerJ. 2021 Jul 27;9:e11750. doi: 10.7717/peerj.11750 (PMC8323605; doi:10.7717/peerj.11750)
Supplement: Supplemental Information 1 [file peerj-09-11750-s001.docx]

Supplementary Table S1

Table S1. Tracking performance comparison among the evaluated systems testing on zebrafish time-lapse video dataset

| Video ID | MOTP (pixels) | | | | MOTA (1) | | | |
| --- | --- | --- | --- | --- | --- | --- | --- | --- |
|  | MSBOTS | Simple- Tracker | idTracker | Loli Track | MSBOTS | Simple- Tracker | idTracker | Loli Track |
| 1 | 6.346 | 6.124 | 11.388 | 11.662 | 0.988 | 0.910 | 0.976 | 0.998 |
| 2 | 15.024 | 9.339 | 21.434 | 18.395 | 0.893 | 0.805 | 0.526 | 0.991 |
| 3 | 8.113 | 7.725 | 20.648 | 18.854 | 0.998 | 0.483 | 1 | 0.995 |
| 4 | 10.669 | 14.452 | 16.728 | 23.208 | 0.993 | 0.490 | 0.987 | 0.987 |
| 5 | 15.525 | 17.168 | 21.545 | 21.890 | 0.994 | 0.890 | -0.0121 | 0.139 |
| 6 | 12.786 | 17.168 | 13.151 | 15.020 | 0.936 | -0.1247 | -0.2674 | 0.984 |
| 7 | 30.082 | 17.842 | 25.230 | 80.630 | 0.954 | 0.623 | 0.005 | 0.504 |
| 8 | 36.901 | 54.840 | 53.096 | 98.936 | 0.956 | 0.828 | -0.3848 | 0.209 |
| 9 | 15.960 | 15.960 | 29.921 | 142.834 | 0.989 | 0.968 | 0.632 | 0.327 |
| 10 | 25.121 | 25.121 | 219.329 | 189.975 | 0.880 | 0.589 | -0.1812 | 0.986 |
| Average | **17.653** | 18.574 | 43.247 | 62.140 | **0.958** | 0.646 | 0.328 | 0.712 |
| Confidence Interval (95%) | [11.50, 23.80] | [9.95, 27.20] | [4.22, 82.28] | [22.81, 101.47] | [0.93, 0.98] | [0.45,  0.85] | [-0.02, 0.67] | [0.48, 0.94] |
