# Supplementary material for: MSBOTS: a multiple small biological organism tracking system robust against non-ideal detection and segmentation conditions
Source: PeerJ. 2021 Jul 27;9:e11750. doi: 10.7717/peerj.11750 (PMC8323605; doi:10.7717/peerj.11750)
Supplement: Supplemental Information 2 [file peerj-09-11750-s002.docx]

Supplementary Table S2

Table S2. Tracking performance comparison among the evaluated systems testing on artemia

time-lapse video dataset

| Video ID | MOTP (pixels) | | | | MOTA (1) | | | |
| --- | --- | --- | --- | --- | --- | --- | --- | --- |
|  | MSBOTS | Simpl-e Tracker | idTracker | Loli Track | MSBOTS | Simple- Tracker | idTracker | Loli Track |
| 1 | 33.083 | 119.988 | 103.886 | 132.244 | 0.914 | 0.886 | 0.662 | 0.953 |
| 2 | 41.182 | 38.010 | 100.678 | 184.046 | 0.925 | 0.853 | 0.756 | 1 |
| 3 | 23.845 | 92.254 | 3.014 | 142.122 | 0.714 | 0.456 | 0.705 | 0.925 |
| 4 | 32.671 | 78.845 | 4.103 | 126.743 | 0.732 | 0.405 | 0.617 | 0.815 |
| 5 | 30.878 | 96.625 | 4.767 | 136.148 | 0.707 | 0.355 | 0.819 | 0.871 |
| Average | **32.332** | 85.144 | 43.290 | 144.261 | 0.798 | 0.591 | 0.712 | **0.913** |
| Confidence Interval (95%) | [26.91, 37.76] | [58.64, 111.65] | [-3.93,  90.51] | [124.15, 164.37] | [0.70, 0.90] | [0.37, 0.82] | [ 0.64, 0.78] | [0.85, 0.98] |
