# Supplementary material for: MSBOTS: a multiple small biological organism tracking system robust against non-ideal detection and segmentation conditions
Source: PeerJ. 2021 Jul 27;9:e11750. doi: 10.7717/peerj.11750 (PMC8323605; doi:10.7717/peerj.11750)
Supplement: Supplemental Information 3 [file peerj-09-11750-s003.docx]

Supplementary Table S3

Table S3. Tracking performance comparison among the evaluated systems testing on daphnia time-lapse video dataset

| Video ID | MOTP (pixels) | | | | MOTA (1) | | | |
| --- | --- | --- | --- | --- | --- | --- | --- | --- |
|  | MSBOTS | Simple- Tracker | idTracker | Loli Track | MSBOTS | Simple- Tracker | idTracker | Loli Track |
| 1 | 5.191 | 4.834 | 3.936 | 9.067 | 0.932 | 0.630 | 0.742 | 0.945 |
| 2 | 8.766 | 63.723 | 3.428 | 57.848 | 0.969 | 0.243 | 0.921 | 0.867 |
| 3 | 10.012 | 59.157 | 1.573 | 36.289 | 0.987 | 0.548 | 0.988 | 0.800 |
| 4 | 36.072 | 135.218 | 38.169 | 120.587 | 0.870 | 0.683 | 0.869 | 0.596 |
| 5 | 30.293 | 68.332 | 31.484 | 86.115 | 0.930 | 0.564 | 0.894 | 0.845 |
| Average | 18.067 | 66.253 | **15.718** | 61.981 | **0.938** | 0.534 | 0.883 | 0.811 |
| Confidence Interval (95%) | [5.74, 30.39] | [25.63, 106.87] | [0.27,  31.17] | [24.04, 99.92] | [0.90, 0.98] | [0.38, 0.68] | [0.80, 0.96] | [0.70, 0.93] |
